# Case of Lemierre's Syndrome Presenting With Neuro-Ophthalmologic Complications That Worsened After Stopping Corticosteroids

**DOI:** 10.7759/cureus.53640

**Published:** 2024-02-05

**Authors:** Aseel Abuhammad, Osayd Nassar, Mohammed G Tomizi, Khadeejeh Alfroukh, Abdelwadod Abuturki

**Affiliations:** 1 Faculty of Medicine, Al-Quds University, Jerusalem, PSE; 2 Department of Internal Medicine, Al-Ahli Hospital, Hebron, PSE

**Keywords:** case report, oropharyngeal infection, jugular vein thrombosis, cranial nerve palsies, lemierre's syndrome

## Abstract

Lemierre's syndrome (LS) is a rare disorder that manifests as septic internal jugular thrombophlebitis following a recent oropharyngeal infection. This article details a unique case of LS, where the patient presented to the emergency room with complaints of vomiting, headache, diplopia, and left eye pain. Due to a history of sore throat, headache, neck pain, fever, and nausea five days prior to admission, the patient was initially treated with amoxicillin/clavulanate for suspected tonsillitis. A positive meningeal sign and elevated temperature were observed during the clinical examination. Lumbar puncture (LP) was deferred based on imaging indicating potential increased intracranial pressure (ICP). Nevertheless, the patient received vancomycin, ceftriaxone, and dexamethasone as an initial course of treatment for presumed bacterial meningitis. Significant improvement was observed within the first four days of admission, with no subsequent episodes of fever, nausea, or headache. However, upon discontinuation of corticosteroid therapy, the patient experienced severe headaches and frequent vomiting. An urgent brain CT scan confirmed the extension of the left internal jugular vein (IJV) thrombosis to the ipsilateral sigmoid sinuses. Metronidazole and anticoagulant medication were initiated upon LS diagnosis. There is a paucity of discussions on corticosteroid use in LS, with no definitive statistics in the current literature. This case underscores the importance of recognizing and effectively managing interconnected clinical manifestations.

## Introduction

Lemierre's syndrome (LS) is an uncommon condition that usually develops after an oropharynx-related infection. Internal jugular vein (IJV) thrombophlebitis, septic emboli, and metastatic abscesses are its hallmarks [1]. It can affect different organs and present in diverse ways, such as respiratory failure [1]. LS is regarded as an uncommon disease owing to its incidence of 1/1,000,000 [1]. Ophthalmologic signs are a rare and unusual presentation of LS [2]. Although anticoagulant drugs and antibiotic therapy have both been mentioned as potential treatments for LS in several studies, no ideal treatment plan has been identified [1]. In this article, we present a case of LS that included ptosis, extraocular motility disorder (diplopia, cranial nerve (CN) III/IV/VI palsies, ophthalmoplegia, and nystagmus), mydriasis, papilledema, and headache after an oropharyngeal infection. This combination of symptoms has only occasionally been documented. Additionally, after receiving steroid treatment, our case was improving, but after the steroids were stopped, she developed rebound phenomena.

## Case presentation

A 15-year-old female patient with no past medical history presented to the ED with left eye pain, diplopia, headache, and vomiting. She had previously been treated with ceftriaxone, amoxicillin/clavulanate, and paracetamol but showed little improvement after the tonsillitis diagnosis. She had a sore throat, headache, neck pain, fever, and nausea for the previous five days. 

On examination, the patient was well, alert, and had normal vital signs, except her temperature was 102.2°F. Further clinical evaluations revealed neck rigidity and a hyperemic pharynx with a positive Kernig's sign. Left eye ptosis, a dilated non-reactive left pupil, failure of the left eye to move laterally, and horizontal nystagmus were all noticeable during the physical examination of the eye. A bilateral papilledema was discovered during her fundus exam. The review of other systems was uneventful. 

**Figure 1:**
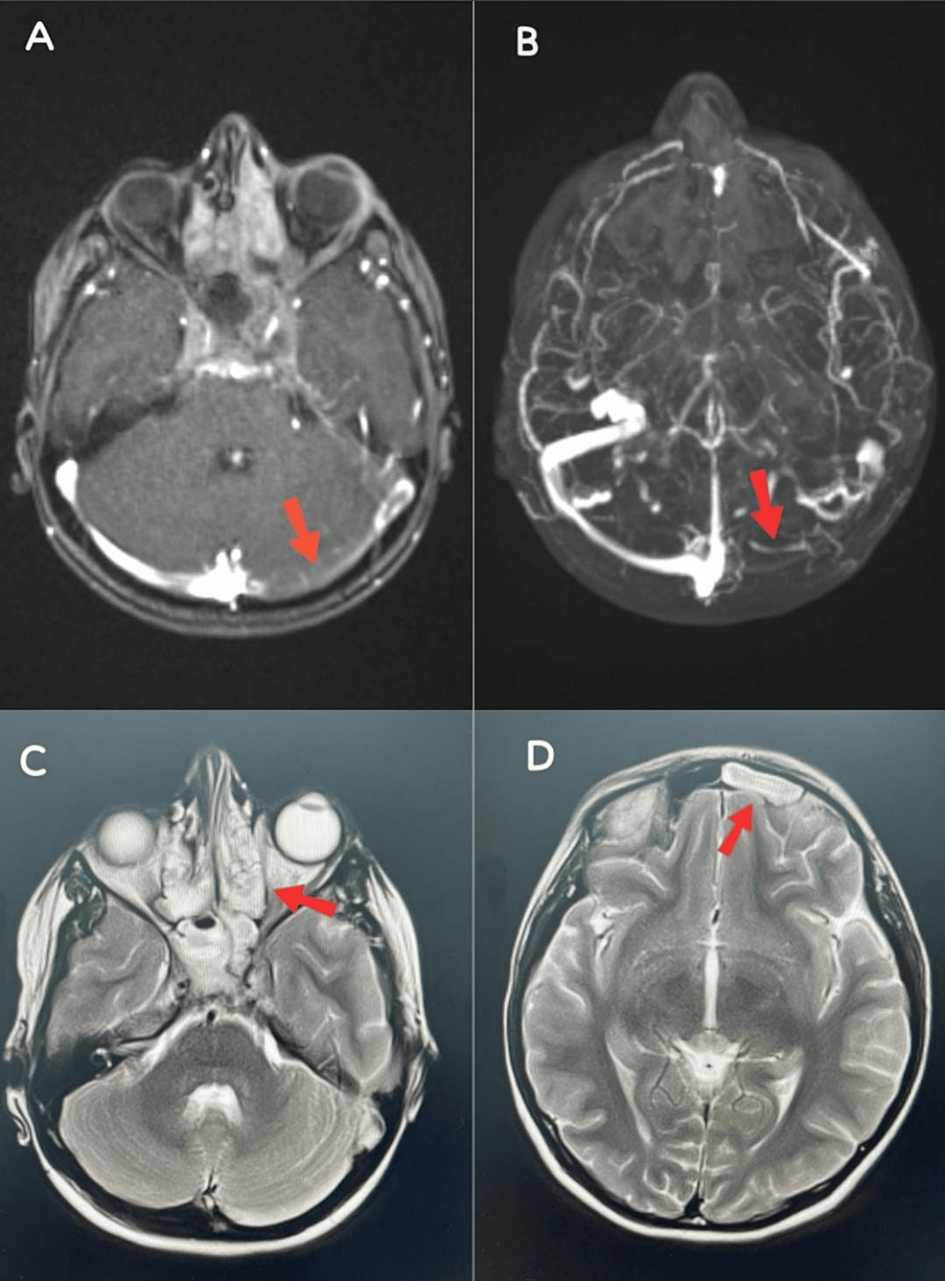
Head magnetic resonance imaging. (A-B) Sections show left internal jugular vein thrombosis (red arrows); (C-D) sections show complete opacification of the sinuses with evidence of diffusion restriction and enhancing sinus walls, indicating severe panniculitis (red arrows).

A brain MRI with IV contrast was done, and the results revealed significant pansinusitis and left IJV/sigmoid sinus thrombosis (Figure 1). The mild effacement of the peri-optic CSF space and minimal tortuosity of both optic nerves may have been caused by papilledema. High levels of erythrocyte sedimentation rate, C-reactive protein (CRP), and WBCs were found in the initial laboratory investigations. Blood, throat, and urine cultures revealed no growth and were all negative. Lumbar puncture (LP) was avoided because of elevated intracranial pressure (ICP). However, as an initial course of treatment for presumed bacterial meningitis, the patient was started on vancomycin, ceftriaxone, enoxaparin, and dexamethasone.

**Figure 2:**
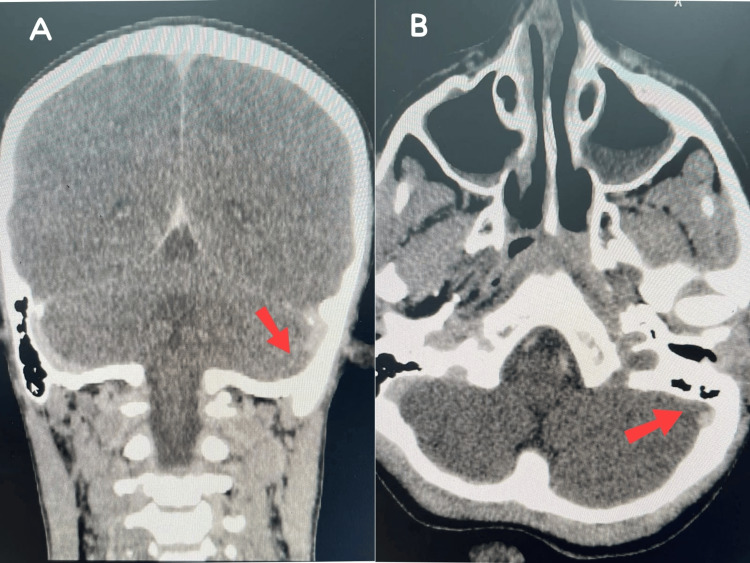
Head CT scan with IV contrast showing a filling defect within the left internal jugular vein extending to the ipsilateral sigmoid sinuses (A-B). (A) Section indicating sigmoid sinus thrombosis; (B) Section showing left internal jugular vein thrombosis.

The patient significantly improved over the first four days after being admitted, with no new episodes of fever, vomiting, or headache noted. Her sixth-nerve palsy persisted despite an examination revealing no ptosis. Additionally, the CRP level dropped to 31. Dexamethasone was terminated after a total of four days, and the other management was continued. Unfortunately, she developed severe headaches and frequently vomited. The filling defect detected within the left IJV extending to the ipsilateral sigmoid sinuses was confirmed by an urgent brain CT scan with IV contrast, in keeping with known venous thrombosis (Figure 2). In addition, there was distension of the left cavernous sinus by high-density material, indicating left cavernous sinus thrombosis.

After that, she was started on metronidazole for 10 days. The patient was discharged on warfarin after being admitted for 12 days. When a follow-up was conducted two weeks later, the patient was in good health, with her symptoms resolved, no history of recurrence, and inflammatory markers remaining within the normal range.

## Discussion

Ocular symptoms of LS are infrequently documented and can occur with unusual characteristics [2]. We found it interesting that one of the publications reviewed the literature up to 2019 and found a total of 27 individuals with LS who presented with ophthalmologic signs [2]. Extraocular motility dysfunction, including diplopia, cranial nerve (CN) III (oculomotor), IV (trochlear), and VI (abducens) palsies, ophthalmoplegia, and strabismus, was the most prevalent ocular symptom found. These patients had a 44% incidence rate of abducens nerve palsy, which was significantly high. A total of 70.37% of cases were reported to have cavernous sinus thrombosis. Ptosis (33% of individuals) was the second-most prevalent ocular symptom reported. In 11 cases, visual acuity was impaired, and in three of them, the impairment was significant [2]. In another study, 27 people with LS and ophthalmic problems were discovered internationally between 2000 and 2017. Of these patients, 35% suffered from decreased vision, 38% had periocular edema, 28% showed ptosis, and 28% reported extraocular motility disorder. Cerebral vein (70%) and ophthalmic vein (55%) thrombosis, as well as septic embolism (7%), periorbital cellulitis/orbital abscess (2%), and carotid artery stenosis (14%), were the most common causes of symptoms [3].

Cavernous sinus thrombosis (CST) can cause a variety of symptoms, including proptosis, CN III (oculomotor), IV (trochlear), and VI (abducens) palsies, chemosis, and high-grade fever. It can also cause headaches. Furthermore, sinus thrombosis may decrease CSF flow, resulting in elevated ICP and papilledema. These two causes, ICP linked to cavernous sinus thrombosis and abducens nerve palsies, can explain the impairment of the abducens nerve. CST may also cause paralysis and inflammation of the lateral rectus muscle, affecting the abducens nerve [2].

In order to effectively treat people with LS, a combination of disciplines is required. To arrive at a quick diagnosis and achieve a successful therapeutic outcome, teamwork among infectious disease specialists, pharmacists, radiologists, otolaryngologists, and surgeons is crucial. Using the proper medications and performing surgical drainage of the abscess formation are part of the treatment. Because β-lactamase-producing strains of *F. necrophorum* have been described and co-infecting bacteria may also produce β-lactamases, any antibiotic regimen should include a β-lactamase inhibitor. According to one study, metronidazole is the antibacterial medication most frequently recommended [4]. Metronidazole has high uptake into most tissues, including cerebral spinal fluid, and its bioavailability is not affected by oral administration [4]. In the present case, *F. necrophorum* cultures were not available in the hospital, but the patient responded well to metronidazole.

Steroids have not been thoroughly researched in the management of LS, and there is no conclusive evidence that they have a positive impact [5]. However, in some case reports, the administration of steroids was found to significantly improve the condition. We found one case report of a 73-year-old male patient diagnosed with LS, clivus osteomyelitis, and inflammatory granuloma in the cavernous sinus-suprasellar region. Steroids and antibiotics were both used in his treatment. As a result, his symptoms of ocular disorder, headache, and fever significantly improved [6]. There were also instances of LS associated with various complications that required the prescription of steroids; one of these cases was a retropharyngeal abscess that significantly improved with dexamethasone [7]. Additionally, LS in systemic lupus erythematosus (SLE) patients has been documented and treated with IV methylprednisolone [8]. In another article, inflammatory phlebitis with bilateral inflammatory constriction of the internal carotid arteries required steroid administration with a favorable response to treatment [9].

In this particular case, there were a variety of features. First, in addition to various ophthalmologic complications, our patient had meningitis-related positive signs and symptoms. Second, the patient was successfully treated for four days as if they had meningitis with steroids and antibiotics; however, as soon as the medication was discontinued, the patient's condition deteriorated, and the thrombosis deepened. Corticosteroids may be deemed beneficial for these patients in light of this treatment plan and the lack of knowledge available about the management of LS.

## Conclusions

The utilization of corticosteroids in LS cases remains an understudied area, with a lack of conclusive data on their application in the existing literature. Our specific case demonstrated notable clinical amelioration following corticosteroid administration. However, upon cessation of the medication, a recurrence of symptoms ensued, accompanied by the identification of new thrombotic occurrences. Consequently, further research is imperative to refine and tailor patient care. This particular incident underscores the critical significance of recognizing and proficiently addressing interconnected clinical manifestations.
